# Development and characterization of a novel B7-H3 rabbit monoclonal antibody for glioma diagnosis

**DOI:** 10.3389/fphar.2026.1736583

**Published:** 2026-01-23

**Authors:** Yue Li, Zhihong Wang, Qian Wen, Menghsuen Chiu, Xian Jia, Yijin Li, Xiaoyun Shang

**Affiliations:** 1 Chongqing University Fuling Hospital, Chongqing, China; 2 T-Maximum Pharmaceutical, Co., Ltd, Chongqing, China; 3 T-Maximum Pharmaceutical, Inc., Delaware, United States; 4 T- Maximum Pharmaceutical, Co. Ltd, Ningbo, China; 5 Chongqing International Institute for Immunology, Chongqing, China

**Keywords:** antibody, B7-H3 (CD276), diagnosis, glioma, immune checkpoint, immunohistochemistry, tonsil, vascular

## Abstract

**Background:**

Glioma is the most common primary brain tumor of the central nervous system and is associated with poor clinical outcomes, particularly in high‐grade diease. The immune checkpoint protein B7‐H3 (CD276) is significantly overexpressed in glioma and multiple other solid tumors, highlighting its potential as a diagnostic biomarker and therapeutic target. However, the availability of high‐performance antibodies suitable for clinic in vitro diagnosis (IVD) applications remains limited.

**Objective:**

This study aimed to develop a novel rabbit monoclonal antibody (clone 36H7) targeting human B7‐H3, and to evaluate its suitability for clinical diagnostic use.

**Methods:**

Rabbits were immunized with recombinant human B7‐H3 protein, followed by monoclonal antibody generation and systematic screening. The resulting antibody was assessed for specificity, affinity and stability. Its performance was validated through Western blot, ELISA, surface plasmon resonance (SPR), flow cytometry, immunofluorescence, and immunohistochemistry (IHC).

**Results:**

Clone 36H7 demonstrated high specificity and robust stability across multiple assay platforms. In IHC analysis of glioma samples (n = 206), B7‐H3 positivity was detected in 97% of cases. In addition, B7‐H3 expression was observed in 85.4% of vascular endothelial cells, supporting its strong detection capability in tumor‐associated compartments. Tonsil tissue was established as a reliable quality control material to ensure assay consistency and reproducibility.

**Conclusion:**

The rabbit monoclonal antibody 36H7 exhibits excellent specificity, stability and board applicability across analytical platforams, meeting key requirments for clinical diagnostic development targeting B7‐H3 in glioma.

## 1 Introduction

Glioma, an aggressive malignancy, accounts for approximately 49% of primary intracranial tumors (Schaff and Mellinghoff, 2023), with high-grade gliomas constituting 90% of these cases (Luo et al., 2023). Current diagnostic methods such as magnetic resonance imaging (MRI) (Satragno et al., 2024) and tumor tissue biopsy are critical for tumor grading and personalized treatment planning. Immunohistochemistry (IHC) detection and genetic testing play pivotal roles in identifying therapeutic targets, such as IDH1 (Fan et al., 2020) mutation or VEGF (Olafson et al., 2019; Chiu et al., 2023) which guide treatment with agents like ivosidenib or bevacizumab. For immunotherapy, B7-H3 (CD276) (Santiago-Vicente et al., 2024) and IL13Ra2 (Zhang et al., 2024) has emerged as a promising target for CAR-T therapy and other interventions. B7-H3 (CD276), an immune checkpoint protein of the B7 family (Aggarwal et al., 2022), is characterized by a 4Ig-2Ig-like domain structure in human and 2Ig-like domain in mice (Steinberger et al., 2004; Getu et al., 2023). It is a type I transmembrane glycoprotein encoded on chromosome 15 (Castellanos et al., 2017), it comprises 534 amino acids, with a topological structure consisting of an extracellular domain of amino acids 29 to 466, a helical transmembrane region from amino acid 467 to 487, and a cytoplasmic domain extending from 488 to 534. Its expression is predominantly observed in various tumors, including glioblastoma (Zhang et al., 2019), mesothelioma (Matsumura et al., 2020), breast cancer (Avci et al., 2022), small cell lung cancer (Carvajal-Hausdorf et al., 2019), colon cancer (Gao et al., 2021), and other solid tumors, but absent in healthy brain tissues.

The expression of B7-H3 varies across tumor types and it is frequently upregulated in a broad range of malignancies and has been associated with aggressive tumor behavior and unfavorable clinical outcomes (Zhou and Jin, 2021). Notably, B7-H3 is not expressed in healthy brain tissue but is highly overexpressed in gliomas (Zhou et al., 2013), making it an ideal immunotherapy target, particularly for CAR-T therapy. Accordingly, patients with positive B7-H3 immunohistochemical staining are likely to benefit from B7-H3-targeted therapies. However, the clinical utility of B7-H3 in diagnostics is limited by the lack of specific antibodies capable of accurately detecting its expression without false-positive staining of healthy tissues. Therefore, the development and rigorous validation of highly specific anti-B7-H3 antibodies is essential to enable accurate assessment of B7-H3 expression in tumors and to support its reliable use as both a diagnostic biomarker and a companion tool for B7-H3-targeted therapies.

In immunohistochemical analyses of tumor paraffin-embedded slides, it is challenging to ensure complete consistency in staining and detection conditions across different laboratories or pathology departments. The use of quality control (QC) slides enables the exclusion of result variability arising from technical differences in experimental procedures. When identical QC slides are applied across laboratories and consistently reproduce their expected staining characteristics, inter-laboratory variability can be considered unlikely to affect the interpretation of sample results. Therefore, the selection of appropriate QC slides is critical for ensuring the reliability and comparability of immunohistochemical assessments. The ideal control tissue should exhibit consistent target antigen expression and closely resemble the patient’s diseased tissue. Given the variable expression of the B7-H3 protein target in tumor tissues (Deng et al., 2020), identifying a stable and easily accessible control tissue is essential. While most healthy tissues exhibit low level B7-H3 protein expression (Li et al., 2024), some lymphoid tissues such as the tonsil, have been reported to express B7-H3 protein (Chen et al., 2020). Following rigorous evaluation, we identified tonsil tissues as a reliable control for B7-H3 IHC analysis.

The emergence of CAR-T therapies (Li et al., 2022) tarting B7-H3 has emphasized the critical need for robust and analytically validated assays capable of accurately assessing B7-H3 expression in tumor specimens. Despite the demand, no FDA-approved companion diagnostic assay for B7-H3 is current avaliable. Among commercially available research-grade rabbit monoclonal antibodies against B7-H3, only a minority exhibit compatibility across multiple analytical platforms. For instance, the anti-CD276 antibody clone EPNCIR122 is not suitable for IHC-P, and several widely used clones, including SP206, EPR20115, and D9M2L, are predominantly recommended for Western blotting and IHC-P. Moreover, information from the Abcam and Cell Signaling Technology indicates that few antibodies demonstrate consistent and high-quality performance across diverse modalities.

In contrast, the newly developed rabbit monoclonal antibody clone 36H7 exhibits high specificity and broad utility across multiple platforms, including flow cytometry, immunohistochemistry, immunofluorescence, Western blotting, and ELISA. Given the objective of advancing 36H7 toward clinical deployment as a companion diagnostic reagent, prioritization of a single primary detection modality is necessary. Immunohistochemistry was selected based on its clinical relevance and diagnostic utility across the tumor types of interest, while still permitting future development of non-IHC-based assays. SP206 and D9M2L were selected as control antibodies in this study, based on its clinical relevance and diagnostic utility across the tumor types of interest.

During the development of this novel antibody, we employed a single B cell-based antibody discovery approach, which significantly reduced the antibody development timeline compared to conventional hybridoma technology, while yielding antibodies with superior affinity and specificity. Furthermore, recombinant cloning techniques were utilized to construct antibody sequences into expression plasmids, which were subsequently transfected into engineered host cells for antibody production. This strategy ensures consistent antibody expression and minimizes batch-to-batch variability. Through a series of stringent screening and validation steps, we successfully identified the monoclonal antibody 36H7, which exhibits the desired binding profile and functional characteristics required for our applications.

Rigorous validation demonstrated its exceptional performance in detecting B7-H3 in glioma and associated vascular endothelial cells. These results not only highlight the clinical diagnostic potential of this antibody but also provide critical insights into B7-H3 expression patterns, supporting its role in glioma diagnosis and treatment planning. This work fills an unmet need for reliable diagnostic tools, paving the way for improved glioma detection and precision therapy.

## 2 Materials and methods

### 2.1 Tissue specimens and cell lines

The tissue specimens used in this study included formalin-fixed paraffin-embedded (FFPE) tissues, tissue microarrays (TMA) and cells. The FFPE blocks of tonsil were obtained from the archive of the Pathology Department of Chongqing University Fuling Hospital. The tonsils removed were in a healthy state. Patient elected to undergo a tonsillectomy to alleviate chronic snoring that was impairing sleep quality and to mitigate the risk of airway obstruction caused by enlarged tonsils. Tumor tissues were sourced from bioaitech company (Xian, China). The slides of glioma were from the first affiliated hospital of Soochow university. TMA were purchased from Taize Biotechnology (Guangzhou, China). The TMA comprised 37 different types of healthy human organs dots, with each tissue core measuring 1.5 mm in diameter. U251 and Jurkat cell lines were purchased from National Collection of Authenticated Cell Cultures.

### 2.2 Antibodies

The B7-H3 antibody employed in this study is clone 36H7. New Zealand white rabbits were immunized with the B7-H3 antigen (amino acids 29–245, the extracellular domain and was supplied in a non-denatured form, with activity confirmed by ELISA), followed by spleen isolation and preparation into a single-cell suspension. After removing red blood cells, further screening was conducted. B cells were fluorescently labeled and sorted using flow cytometry, and individual B cells were cultured in 96-well plates. Supernatants from 42 cloned cells were selected for ELISA testing, and 12 positive clones were identified. Ten candidate cells were expanded in culture, and after confirming positivity through flow cytometry, IHC experiments were performed on cell smears to screen out clone 36H7 from the 10 candidate clones. The B cells of clone 36H7 were sequenced to obtain the light chain and heavy chain sequences. This part of the work was outsourced to Dima Biotechnology (Wuhan, China). The antibody of 36H7 was manufactured by ACROBiosystems (B73-M702). A pcDNA3.4 vector was selected for the expression of the antibody in human 293 cells (HEK293), and the purification was done using a Protein A column. Additional B7-H3 antibody clones were purchased from Cell Signaling Technology (B7-H3 D9M2L) and Abcam (B7-H3 SP206). B7-H3 antigen was purchased from ACROBiosystems (B73-H52E2). Detailed information of the antibodies is provided in Table 1.

**TABLE 1:** The information about antibodies and antigen.

Antibodies and antigen	Catalog	Manufacturer
B7-H3(36H7)	B73-M702	ACROBiosystems
Anti-CD276 [SP206]	ab227670	Abcam
B7-H3 (D9M2L) XP® rabbit mAb	14058	Cell signaling technology
Isotype rabbit IgG	BX60003B-C3	Biolynx
CR2/CD21 rabbit mAb	A22401	Abclonal
Anti-VEGF receptor 2	ab115805	Abcam
Goat anti-rabbit IgG (H + L) cross-adsorbed secondary antibody, HRP	G-21234	Invitrogen
Secondary antibody, HRP	IBF10-01	Accupath
Secondary antibody, HRP	PV-9001	ZSGB-BIO
Human B7-H3/CD276 protein, His Tag (MALS verified)	B73-H52E2	ACROBiosystems

### 2.3 Detection method of antigen-blocking assay

The B7-H3 protein was used as a blocker for the B7-H3 (36H7) antibody. For immunohistochemistry (IHC) assay, the B7-H3 protein was added to the B7-H3 (36H7) antibody solution to achieve final concentrations of 0 μg/mL, 0.05 μg/mL, 0.1 μg/mL, and 0.5 μg/mL, respectively. Subsequently, the protein-antibody mixture was incubated on antigen-retrieved tissue slides for 40 min at room temperature. After washing, the slides were incubated with polymer (secondary antibody) for 2 min at room temperature, followed by incubation with 3, 3′-diaminobenzidine (DAB) for 8 min and hematoxylin for 8 min at room temperature. Finally, the slides were dehydrated and mounted with neutral resin.

### 2.4 Surface plasmon resonance

The affinity of anti-B7-H3 antibody against the antigen was measured by surface plasmon resonance technology. Protein A Chip (Cytiva: 29127556) was used to capture the antibody, 1 μg/mL antibody in Running Buffer, 10 μL/min. The flow-through antigen concentration ranged from 1.5 nM to 100 nM, 10 μL/min. Measurements were performed using Biacore 8K instrument (Cytiva: 29337763).

### 2.5 Western blot

Cells were lysed with cell lysis buffer, and the lysates were denatured for 10 min at 95 °C with SDS-PAGE sample loading buffer. The samples were then electrophoresed on 10% SDS-PAGE gels and transferred to polyvinylidene difluoride (PVDF) membranes (B7-H3 protein: 5 ug, U251 cell lysate: 20 μg, Jurkat cell lysate: 20 μg).The membranes were blocked with 5% nonfat milk in TBST [20 mM Tris-HCl (pH 7.5), 150 mM NaCl, and 0.05% (v/v) Tween 20] and incubated with B7-H3 antibody (0.5 μg/mL) for 15 h at 4 °C. After thorough washing, the blots were incubated with goat anti-rabbit IgG conjugated with horseradish peroxidase (HRP); the dilution ratio was 1:1000. Protein bands were visualized using TMB chromogenic solution, and images were acquired using a fluorescence imaging system (Vilber Fusion FX6). The experiment was repeated three times.

### 2.6 Enzyme-linked immunosorbent assay (ELISA)

96-well plates were coated with 1 μg/mL B7-H3 protein solution (100 μL per well) and incubated overnight at 4 °C. The plate was washed with phosphate buffer solution (PBS) before each addition of reagents. After being blocked with 3% Bovine Serum Albumin (BSA) for 1.5 h, B7-H3 antibody at concentrations ranging from 0.12 ng/mL to 62.50 ng/mL was added and incubated for 1.5 h at 37 °C. Horseradish peroxidase (HRP)-conjugated anti-rabbit antibody was added to the wells and incubated for 1 h at 37 °C, followed by the addition of tetramethylbenzidine (TMB) to the wells and incubating at 37 °C for 14 min. The enzymatic reaction was stopped by adding sulfuric acid. The optical density was measured at 450 nm.

### 2.7 Flow cytometry

Flow cytometry experiments were performed using CHO cells that had been verified to express B7-H1, B7-H2, B7-H3, and B7-H4. Cells were incubated with 1 μg/mL B7-H3 antibody at room temperature for 1 h. After washing with PBS, fluorescent secondary antibody was added at room temperature for 1 h. Measurements were performed using FACSCanto II (BD Biosciences, Heidelberg, Germany). Data analysis was performed using FlowJo software.

### 2.8 Immunofluorescence

Immunofluorescence staining was performed using Alexa Fluor 647-conjugated goat anti-rabbit IgG (Thermo Fisher Scientific; A21245) as the secondary antibody. Slides were deparaffinized, subjected to EDTA-based antigen retrieval, quenched for endogenous peroxidase activity, and blocked prior to incubation with the primary antibody B7-H3 (36H7, 1.0 μg/mL) for 40 min at room temperature. The slides were then incubated with the secondary antibody (5.0 μg/mL) for 30 min, nuclear staining with DAPI for 10 min, mounted with antifade medium, and imaged under appropriate fluorescence channels.

The Tyramine Signal Amplification (TSA)-Opal fluorescent detection reagents were obtained from Panova Technology (Beijing, China). The kit was composed of six TSA fluorophores and DAPI. All fluorophores and DAPI were prepared according to the manufacturer’s instructions. The final staining protocol is detailed in Table 2.

**TABLE 2:** The conditions of IF.

Primary Ab	μg/mL	Incubation (min)	Temperature (°C)	Fluorescent
B7-H3(36H7)	1.0	60	25 °C ± 2 °C	570 (TSA)
Anti-VEGF receptor 2**	0.35	30	25 °C ± 2 °C	520 (TSA)
Goat anti-Rabbit	1.0	30	37 °C ± 2 °C	—
CD21*	3.6	60	37 °C ± 2 °C	520
DAPI	1:100 (dilution rate)	10	25 °C ± 2 °C	488

The dilution rate of TSA is 1:100. *This antibody was used on tonsil slide. **This antibody was used on glioma to illustrated vessels

### 2.9 Immunohistochemistry

For immunohistochemistry (IHC), 2–10 μm tissue slides were prepared and stained using the BOND III Leica system. The IHC protocol included baking slides at 60 °C for 30 min, epitope retrieval at 100 °C for 10 min with ER2 (EDTA), peroxide treatment for 15 min, primary antibody incubation for 40 min, polymer application for 2 min, DAB development for 8 min, and hematoxylin counterstaining for 8 min. We also explored manual manipulation, acid retrieval, and different antibody incubation times. Stained slides were scanned using the 3DHISTECH Panoramic MIDI system and analyzed with Slide Viewer software. Micrographs were taken with Nikon ECLIPSE Ei microscope using a ×10 eyepiece and ×10 or ×40 objectives.

### 2.10 Antibody stability testing

To evaluate their suitability for clinical diagnostics applications, antibody stability and functional integrity were assessed under simulated storage stress conditions, including temperature variation and repeated freeze-thaw cycles. Immunohistochemistry (IHC) was employed as the primary modality to characterize and compare antibody performance under these conditions.

Both thermal stability and freeze-thaw stability were tested. For thermal stability, 5 mL of antibody working solution at 1.0 μg/mL was prepared and placed at 35 °C ± 2 °C for 168 h. IHC staining before and after was compared. For freeze-thaw stability, the antibody working solution was frozen at −80 °C ± 2 °C and thawed at 23 °C ± 2 °C. The freeze-thaw process was repeated five times. IHC staining before and after was compared.

### 2.11 Statistical analysis and evaluation of slides staining

Quantification of positive cells in tonsillar tissues sections was performed using the analysis module of SlideViewer software (Ács et al., 2018). Germinal centers, crypt epithelium, and interfollicular regions were manually annotated by a pathologist, after which the software automatically identified and quantified positive stained cells. For each tonsil slides, at minimum 20 germinal centers were analyzed, the assessed crypt epithelial area exceeded 5 mm^2^, and no fewer than 15 interfollicular regions were included. Both the number and proportion of positive cells were recorded and incorporated into subsequent statistical analysis. The data of this study were presented as mean ± standard error. Statistical analysis was performed using ANOVA to determine the significant differences among groups with a threshold set at P < 0.05. The assessment of immunohistochemistry results was accomplished by the Shanghai Histo Pathology Diagnostic Center (Shanghai, China) and the Pathology Department of Chongqing University Fuling Hospital (Chongqing, China).

## 3 Results

### 3.1 Functional verification of B7-H3 (36H7) antibody binding capacity

#### 3.1.1 Determination of the epitope recognition range of the B7-H3 monoclonal antibody 36H7

To determine whether the selected antibody targets the extracellular domain of B7-H3, an antigen-blocking assay was performed using a recombinant protein corresponding to amino acids 29–245 of the human B7-H3 ectodomain.

**FIGURE 1:**
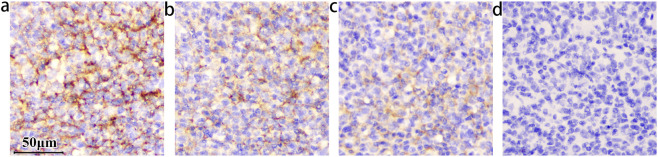
The staining intensity within the germinal center decreased as the concentrations of B7-H3 protein increased. The concentration of the B7-H3(36H7) antibody is 1.0 μg/mL. All of the slides Staining by BOND III. **(a)** No B7-H3 protein added, **(b)** The concentration of B7-H3 protein is 0.05 μg/mL, **(c)** 0.1 μg/mL, **(d)** 0.5 μg/mL. When the concentration is increased to **(d)** 0.5 μg/mL, the antibody binding site is occupied by the B7-H3 protein, which loses the ability to detect the sample. The results confirming the binding specificity of the antibody B7-H3(36H7). scale bar 50 μm. Original magnification, ×400.

As anticipated, increasing concentrations of the recombinant protein induced a dose dependent reduction in staining intensity on FFPE human tonsil section (Figure 1).

This attenuation reflects competitive inhibition, wherein the exogenous recombinant protein occupies the antibody’s complementarity-determining regions, thereby preventing binding to endogenous B7-H3 and resulting in diminished DAB signal intensity. Complete blockade abolished detectable staining. These findings support that the B7-H3 (36H7) antibody recognizes an epitope located within the extracellular region of the B7-H3. However, given that this domain spans 216 amino acids, further studies are required to precisely map the epitope and define the specific amino acid residues recognized by 36H7.

##### 3.1.1.1 Assessment of antibody binding affinity

A qualified antibody should demonstrate adequate binding affinity to its cognate antigen to maintain stable interactions under experimental conditions, thereby ensuring reliable detection through chromogenic or fluorescence-based assays.

**FIGURE 2:**
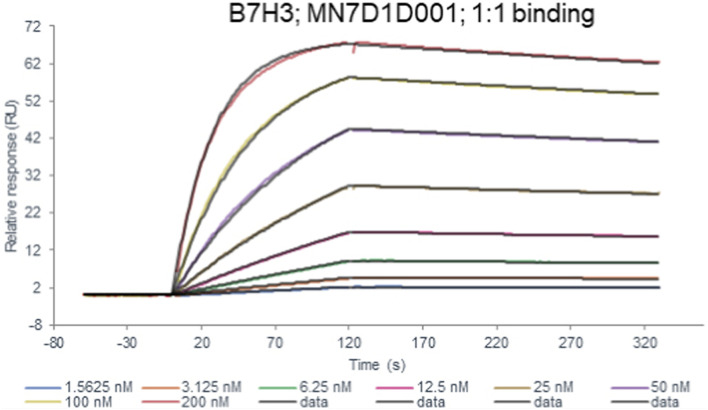
The affinity constant of the B7-H3 peptide binding to the B7-H3(36H7) antibody was measured to be 2.10 × 10^−9^ mol/L. B7-H3(36H7) antibody has a high affinity for antigens.

To evaluate the binding affinity of the B7-H3 (36H7) antibody, surface plasmon resonance imaging (SPRi) analysis was performed. After immobilization of the B7-H3 (36H7) monoclonal antibody on a Protein A sensor chip, serially diluted B7-H3 peptides were injected as analyte over the flow channels for 320s, consisting of a 120s association phase followed by a 200s dissociation phase, yielding a response of approximately 65 resonance units (RU). The overlaid SPR sensorgrams and corresponding fitting curves are shown in Figure 2, illustrating concentration-dependent binding of the B7-H3 peptide. Using a 1:1 binding model, the equilibrium dissociation constant (KD) for the interaction between the B7-H3 peptide and the B7-H3 (36H7) antibody was determined to be 2.10 × 10^−9^ mol/L (Table 3). This nanomolar-range affinity indicates a strong interaction between the antibody and its target antigen.

**TABLE 3:** B7H3(36H7) antibody binding kinetics to B7H3 protein in Biacore kinetic assays.

Chip	Protein A chip
Ligand	B7H3(36H7)
Analyte1 solution	B7H3 protein
Analyte Conc	1.5625–200 nM (2-fold)
1:1 binding ka (1/Ms)	1.83 × 10^5^
kd (1/s)	3.85 × 10^−4^
KD(M)	2.10 × 10^−9^
Fit model	1:1 binding

#### 3.1.2 Specificity of the B7-H3 (36H7) antibody across multiple detection platforms

As described above, most commercially avaliavle rabbit monoclonal antibodies against B7-H3 exhibit limited compatibility across multiple analytical platforms. In contrast, the B7-H3 (36H7) antibody exhibits broad applicability. To characterize its cross-platform performance, the antibody was assessed using Western blotting, ELISA, flow cytometry, immunofluorescence, and immunohistochemistry.

**FIGURE 3:**
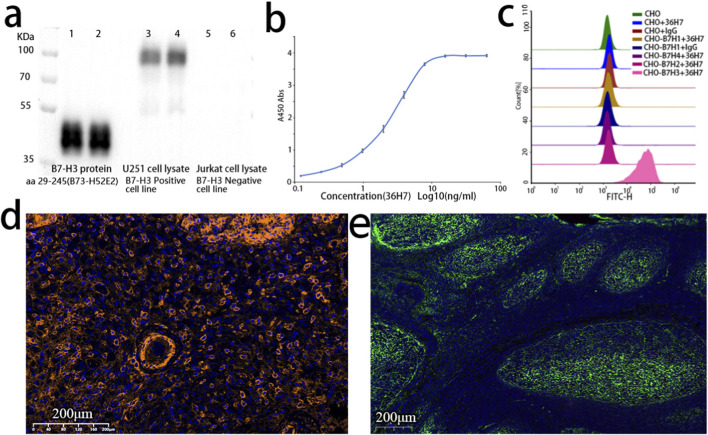
The B7-H3 (36H7) antibody consistently exhibits specific recognition of the B7-H3 antigen across multiple assay platforms. **(a)** Western blot (WB) result of B7-H3 antibody. lane 1 and 2: B7-H3 protein (B73-H52E2 29–245); lane 3 and 4: Lysates of U251 cells; lane 5 and 6: Lysates of Jurkat cells. The 36H7 antibody clone demonstrated specific immunoreactivity with U251 positive control cells, while showing no detectable cross-reactivity with negative cellular components. **(b)** The results of ELISA showed that the B7-H3(36H7) antibody had high sensitivity, and the concentration for 50% of maximal effect (EC50) was 2.474 ng/mL. **(c)** The B7-H3(36H7) antibody does not bind to other proteins within the same family (B7-H1, B7-H2, B7-H4), only binding to B7-H3 protein. **(d)** Fluorescence detection in glioma paraffin-embedded sections using the B7-H3 (36H7) antibody paired with HRP-conjugated goat anti-rabbit secondary antibody (G2-1234) and TSA570 dye. Clear membrane staining is observed. **(e)** Immunofluorescence detection in tonsil paraffin-embedded sections using the B7-H3 (36H7) antibody paired with Alexa Fluor 647-conjugated goat anti-rabbit secondary antibody, showing specific recognition in the germinal centers.

Western blot analysis was conducted using recombinant B7-H3 protein, lysates from the B7-H3-positive U251 cell line (Luther et al., 2010), and lysates from the B7-H3-negative Jurkat cell line (Sun et al., 2020). Consistent with its predicted specificity for the extracellular domain, 36H7 detected a distinct band corresponding to the recombinant B7-H3 protein fragment comprising amino acids 29–245 (Figure 3a). In U251 whole-cell lysates, which contain full-length B7-H3, the antibody detected a band migrating at approximately 100 kDa (Figure 3a), which is consistent with the glycosylated form of full length B7-H3 rather than the theoretical molecular weight of 59 kDa for the 534-amino acid B7-H3 protein (UniProt: Q5ZPR3-1). As expected, no signal was detected in lysates from the B7-H3-negative Jurkat cell line (Figure 3a). Taken together, these results support that the 36H7 antibody specifically recognizes B7-H3 in Western blot assays.

Similarly, the B7-H3 (36H7) antibody exhibited a characteristic sigmoidal response curve in ELISA (Figure 3b), demonstrating concentration-dependent binding to the B7-H3 protein. The concentration required to achieve 50% of the maximal response (EC_50_) was determined to be 2.474 ng/mL.

The B7-H3 (36H7) antibody also exhibited high specificity in flow cytometric analysis. CHO cells expressing other members of the B7 family, including B7-H1, B7-H2, and B7-H4, were used as potential interfering controls. Strong fluorescent signals were observed exclusively in CHO cells expressing B7-H3 (Figure 3c), indicating specific binding of 36H7 to B7-H3 without cross-reactivity to its homologs. CHO cells expressing the other three proteins were confirmed to be positive using the corresponding antibodies (data not shown).

Like most unconjugated antibodies, the B7-H3 (36H7) antibody requires an anti-rabbit secondary antibody to facilitate fluorescent signal generation at antigen sites. Two widely used immunofluorecsence detection strategies were evaluated: direct fluorophore-conjugated secondary antibodies and tyramide signal amplification (TSA). In this study, an Invitrogen fluorophore-conjugated secondary antibody (Thermo Fisher Scientific; A21245) and TSA 570 dye (Panova Technology Beijing, China; 0004100100) in combination with goat anti-rabbit HRP (G-21234) were used for immunofluorescence analysis. Both approaches yielded clear membrane-associated fluorescence signals (Figures 3d,e), consistent with the expected cellular localization of the B7-H3 protein. These results indicate that the B7-H3 (36H7) antibody is compatible with commercially available anti-rabbit secondary antibodies and exhibits specific and robust detection in immunofluorescence assays.

For immunohistochemistry (IHC) analysis, we selected B7-H3 Rabbit Monoclonal Antibody (D9M2L, CST: 14058) and anti-CD276 antibody (SP206, Abcam: ab227670) as benchmarks, both of which are well-established clones suitable for IHC applications and are frequently cited in the literature. Paraffin-embedded tissue samples with reported B7-H3 expression were used, including slides of glioblastoma (Guo et al., 2023), lung adenocarcinoma (Yu et al., 2021), human tonsil (Getu et al., 2023), as well as normal brain tissue.

**FIGURE 4:**
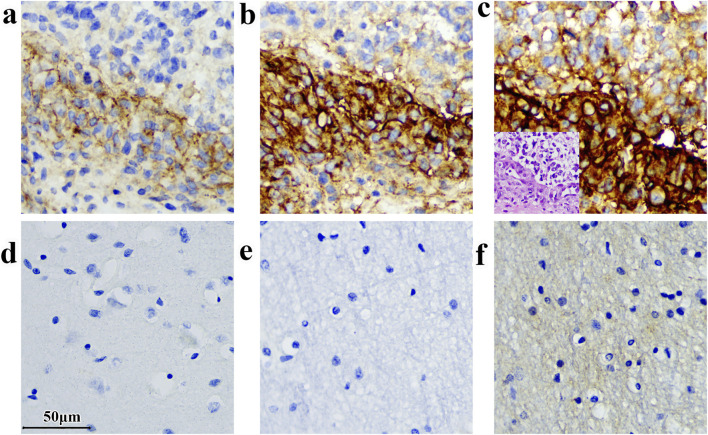
Variations in immunodetection outcomes can arise when distinct antibody clones are employed for analyzing the same sample. **(a)** Glioma sample stained by D9M2L; **(b)** Glioma sample stained by 36H7; **(c)** Glioma sample stained by SP206. The corresponding H&E of glioma sample on the bottom left-hand corner. **(d)** Normal brain tissue stained by D9M2L. **(e)** Normal brain tissue stained by 36H7; **(f)** Normal brain tissue stained by SP206(concentration: 0.5 μg/mL. The staining was performed by BOND III following the method described in section 2.9). Scale bar 50 μm. Original magnification, ×400.

Considering that each clone has an optimal working concentration, adjusting the concentrations of the three antibodies to the same value does not yield the best staining results for each antibody (Supplementary Figure S1). After optimizing the conditions, we determined that a concentration of 0.2 μg/mL (1:50) is suitable for the D9M2L clone, while the working concentration for the SP206 clone is 0.5 μg/mL (1:450), and for the 36H7 clone, it is 1.0 μg/mL. Comparative analyses were subsequently performed on slides of normal brain, glioma, lung adenocarcinoma, and tonsil (Figures 4a–e; Supplementary Figures S1, S2). Among the three antibodies, the SP206 exhibited stronger staining intensity across tissue types. However, while SP206 produced complete membranous staining, it was accompanied by increased background signal, particularly in normal brain slides (Figure 4f), and reducing its concentration to mitigate background led to a loss of staining intensity.

In contrast, the D9M2L clone produced relatively weak staining in glioma and tonsil slides (Figure 4a; Supplementary Figure S2), and increasing its concentration resulted in excessive background staining in lung adenocarcinoma samples. These findings indicate that, individual antibody clones require tailored optimization of staining parameters across tissue types, increasing experimental complexity and resource requirements.

By comparison, the 36H7 clone, used at 1.0 μg/mL, consistently yielded clean background and distinct membranous staining across all tissues examined.This performance simplifies clinical assay workflows and enhance inter-assay consistency.

In summary, the B7-H3 (36H7) antibody consistently exhibits specific antigen recognition across multiple assay platforms, including Western blotting, ELISA, flow cytometry, immunofluorescence, and immunohistochemistry, without detectable cross-reactivity with cellular lysate components or homologous B7 family proteins. Its robust performance in IHC, coupled with consistent staining parameters across diverse tissue types, supports its suitability for clinical diagnostic development.

### 3.2 The antibody 36H7 exhibited excellent stability

To ensure suitability for clinical diagnostics, antibodies must maintain functional activity under potential storage stress condition, including temperature variations and repeated freeze-thaw cycles. Therefore, we evaluated the thermal stability and freeze–thaw resilience of the B7-H3 (36H7) antibody. For the thermal stability test, the antibody was incubated at 35 °C ± 2 °C for 7 days, and for the freeze-thaw assessment, the antibody underwent five cycles of freezing at −80 °C ± 2 °C and thawing at 23 °C ± 2 °C. Antibody activity was then assessed by IHC. B7-H3 (36H7) maintained its ability to recognize the target antigen and showed no non-specific binding under these conditions (Supplementary Figure S3). These results demonstrate that B7-H3 (36H7) possesses the stability required for use as a diagnostic antibody.

### 3.3 Tonsil as a reliable reference tissue for quality assessment

To minimize variability associated with instrumentation and operator handling, standardized quality control slides were implemented. Based on literature review and pathologists consulation, tonsil tissue was selected as the quality control specimen for B7-H3 immunohistochemical staining. Tonsil slides contain both positive and negative regions on the same slide, providing an internal control that supports reliable assessment of B7-H3 staining performance.

**FIGURE 5:**
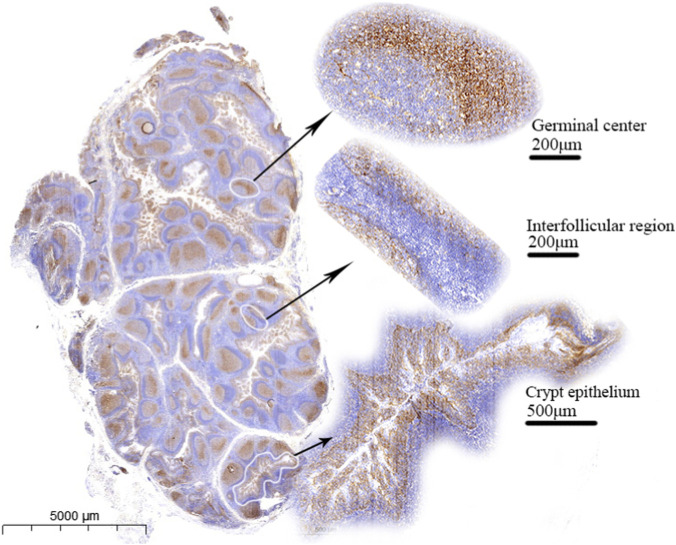
The germinal center and crypt epithelium exhibited positivity for B7-H3 expression, the interfollicular regions were negative.

A total of 50 benign tonsil paraffin-embedded slides obtained from different individuals (20 females and 30 males; median age, 8 years) were evaluated. Immunohistochemical staining with the B7-H3 (36H7) antibody demonstrated positive staining in the germinal centers and crypt epithelium of all tonsil specimens, whereas the interfollicular regions were negative (Figure 5). A significant difference in B7-H3 expression was observed between germinal centers and interfollicular areas (P < 0.001 Supplementary Table S1). The coefficient of variation for the percentage of B7-H3-positive cells within germinal centers was 0.144 (Supplementary Table S1), indicating a stable proportion of positive cells across slides. These results suggest that, provided the intrinsic quality of the tonsil tissue is adequate, consistent staining outcomes can be achieved across pathology laboratories, even when donor age and sex differ. To support pathology technician in assessing tonsil suitability, we identified follicular dendritic cells (FDCs) as the B7-H3-positive cell population within germinal centers (Supplementary Figure S4). Slides lacking well-defined germinal center architecture on hematoxylin and eosin staining or clearly lacking FDCs were deemed unsuitable for use as quality control slides.

**FIGURE 6:**
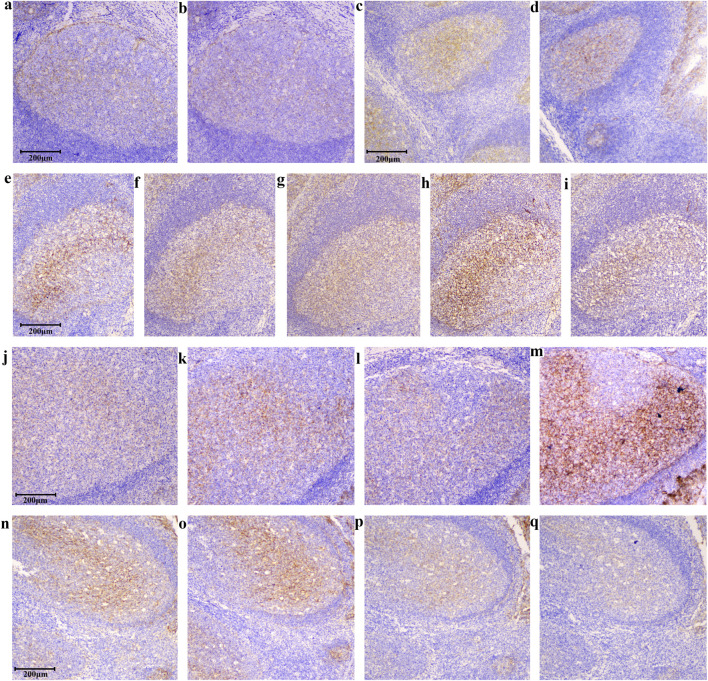
Tests of different staining conditions. Despite variations in IHC conditions, tonsil tissues showed positivity in the germinal center and crypt epithelial and negativity in the interfollicular areas, confirming tonsils as a robust control tissue. scale bar 200 μm, Original magnification, ×100. The results remain consistent across different detection instruments: **(a)** Staining by BOND III (3213972). **(b)** Staining by BOND III (3212863). The slice remained detectable after being stored at −20 °C for 274 days, both Staining by BOND III (3213972): **(c)** Slides were stored for 0 days. **(d)** Slides were stored for 274 days. Differences between manual and automated staining were mainly attributable to the source of the secondary antibody. While staining concentration differs with secondary antibodies from various suppliers, the discrimination between positive and negative areas remains consistent: **(e)** Staining by BOND III (3213972) Epitope retrieval by EDTA, polymer DS9800. **(f)** Staining by manual (Epitope retrieval by EDTA, secondary Ab G-21234). **(g)** Staining by manual (EDTA, secondary Ab PV-9001). **(h)** Staining by manual (EDTA, secondary Ab IBF10-01). Citrate retrieval yields weaker staining than EDTA, while preserving positive detection: **(h)** Staining by manual (EDTA, secondary Ab IBF10-01). **(i)** Staining by manual (citrate pH6.0, secondary Ab IBF10-01). At 10 μm thickness, faint staining appears in negative regions, though the standard range is 2–8 μm: **(j)** 2 μm, **(k)** 4 μm, **(l)** 5 μm, **(m)** 10 μm, Staining by BOND III (3213972). Staining in the positive areas of the tonsil allows for fluctuations in the primary antibody concentration between 0.25 and 2.0 μg/mL: **(n)** 2.0 μg/mL, **(o)** 1.0 μg/mL, **(p)** 0.5 μg/mL, **(q)** 0.25 μg/mL, Staining by BOND III (3213972).

In addition to evaluating variability among tonsil slides, we further examined the robustness of the assay under different testing conditions. Consistene staining results were obtained when assay parameters varied within defined ranges. When the same model of automated staining instrument was used, tonsil slides exhibited nearly identical staining patterns (Figures 6a,b). Although B7-H3 staining remained detectable in slides stored at −20 °C for 274 days (Figures 6c,d), we recommend the use of slides prepared within 3 months to ensure optimal performance. Differences between manual and automated staining were mainly attributable to the secondary antibody reagents; despite minor variations in staining intensity, consistent positivity in germinal centers and crypt epithelium and negativity in interfollicular regions were maintained (Figures 6e–h). EDTA-based antigen retrieval was preferred, as citrate buffer resulted in weaker staining (Figures 6h,i). The optimal section thickness for tonsil slides ranged from 3–6 μm minimizing interpretive challenges from excessive cellular overlap (Figures 6j–m). The 36H7 antibody demonstrated effective staining at 0.5–2.0 μg/mL, with 1.0 μg/mL recommended as the working concentration (Figures 6n–q). Collectively, these findings demonstrate that tonsil tissue serves as a robust and reliable quality control material for B7-H3 immunohistochemistry, capable of maintaining consistent performance across variable assay conditions.

### 3.4 Immunohistochemical testing and pathological evaluation of glioma samples

As a diagnostic antibody, it is essential to clearly define the target specimen type to ensure meaningful clinical interpretation when used in conjunction with targeted therapy. Although positive staining with the B7-H3 (36H7) antibody was also observed in other tumor types (Supplementary Figure S5), glioma FFPE specimens were selected as the primary clinical testing material based on anticipated patient benefit and therapeutic relevance.

Prior to analyzing glioma samples, a normal tissue microarray containing tissue cores from 37 non-neoplastic organs was evaluated to assess baseline B7-H3 expression in healthy tissues (Supplementary Figure S6). The results showed that three tissues-endometrium, pancreas, and liver-exhibited strong staining (These three tissue types lack negative areas and are therefore unsuitable for quality control slides), 13 tissues were negative, and the remaining 21 tissues displayed partial weak staining. The detection results are essentially in line with the previously reported one (Roth et al., 2007).

Immunohistochemistry (IHC) was employed to assess B7-H3 protein expression in FFPE slides from 206 glioma patients, Clinical characteristics of the cohort are summarized in Table 4. Samples were collected between January 2019 and September 2024, with inclusion criteria restricted to clinically confirmed glioma subtypes (glioblastoma, astrocytoma, oligodendroglioma, and diffuse midline glioma) with FFPE processing. Demographic data (gender and age) were unavailable for 42 patients; among the remaining 164 patients, ages ranged from 6 to 80 years (mean, 47.8 years), with a female-to-male ratio of 72:92. Stratification by WHO grade revealed that 35 female and 69 male cases were classified as grade IV, 13 female and 5 male cases as grade III, and 9 cases as intermediate between grades II and III.

**TABLE 4:** Patient characteristics.

Classification	Mean	Range
Age at diagnosis [years]	47.8	6.0–80.0
Sex	n = 164	Median age
Female	72	50.5
Male	92	52
WHO classification	Female [number]	Male [number]
WHO II	5	1
WHO II ∼ WHO III	0	3
WHO III	13	5
WHO IV	35	69

**FIGURE 7:**
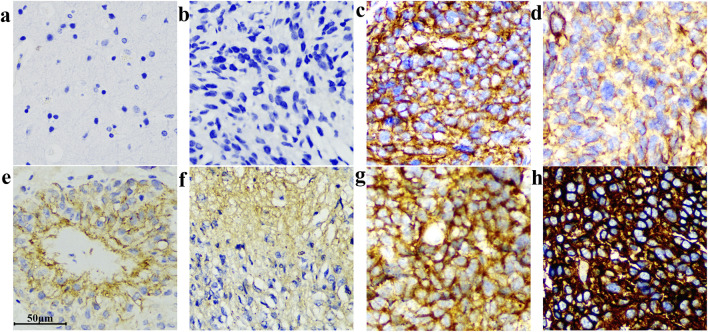
Different IHC staining patterns of B7-H3(36H7) antibody on glioma sections. The antibody demonstrated high sensitivity, enabling robust discrimination between samples with differential target expression levels. **(a)** Normal brain, no background color. **(b)** Negative. **(c)** and **(g)** Strongly positive cell membrane staining. **(d)** Moderately positive cytoplasmic and cell membrane staining. **(e)** and **(f)** Weakly positive cytoplasmic staining. **(h)** Strong positive cytoplasmic and cell membrane staining. Scale bar 50 μm. Original magnification, ×400.

Among 206 cases, 6 cases were B7-H3 protein express negative, 97% of the samples were positive. The negative slides had clean backgrounds and clear cell nuclei. A representative image of the tissue section staining results is shown in Figure 7.

**FIGURE 8:**
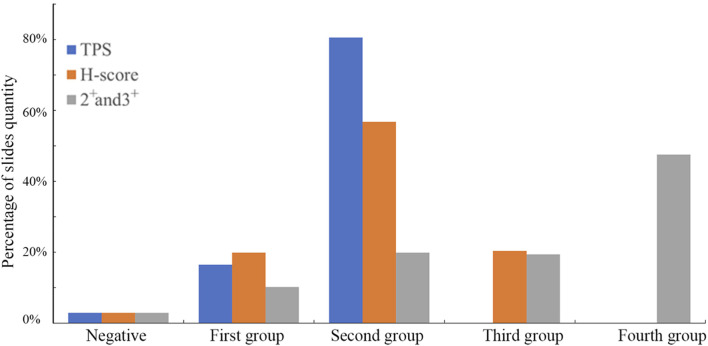
The slides were evaluated using three scoring methods. Different scoring criteria will obtain different results for the same slides, and the patient’s treatment plan needs to be analyzed by the doctor according to the actual situation. TPS has 3 groups, H-score has 4, and 2+ and 3+ method has 5.

Considering the current variability in Immunohistochemistry (IHC) evaluation methods and the fact that this antibody has not yet entered clinical testing, we employed three scoring systems to assess the performance of the B7-H3 (36H7) antibody on tissue slides (Figure 8). The first method categorized the positivity based on the percentage of tumor cells into three levels Tumor Proportion Score (TPS): 0%, 1%–49%, and 50%–100%. The second method classified the results according to the H-score calculation, H-Score = (1 × Percentage of 1^+^) + (2 × Percentage of 2^+^) + (3 × Percentage of 3^+^). 1^+^, 2^+^, 3^+^ indicates the strength of the staining, 1^+^ indicates pale yellow staining, 2^+^ indicates dark yellow staining, 3^+^ indicates dark brown staining Where a H-Score of 0 is considered negative, 1–79 is weak positive, 80–199 is moderate positive, and 200–300 is strong positive. The third method accumulates the percentage of positive cells with staining intensity of 2^+^ and 3^+^. Staining with 1^+^ or 0 is classified as negative, results are divided into five levels: negative, First group 0%, Second group 1%–19%, Third group 20%–49%, and Fourth group 50%–100%.

Each of the three scoring methods offers distinct advantages and limitations. The TPS approach provides straightforward patient stratification but groups all cases with ≥50% positivity together, without accounting for differences in staining intensity. In contrast, the 2^+^/3^+^ percentage-based classification provides finer stratification but requires interpretation by experienced pathologists. The H-Score is widely applicable; however, it lacks flexibility for customization based on drug-specific mechanisms of action. It is important to emphasize that the present discussion pertains only to scoring methodology, and the establishment of clinically relevant cut-off values will require additional investigation.

**FIGURE 9:**
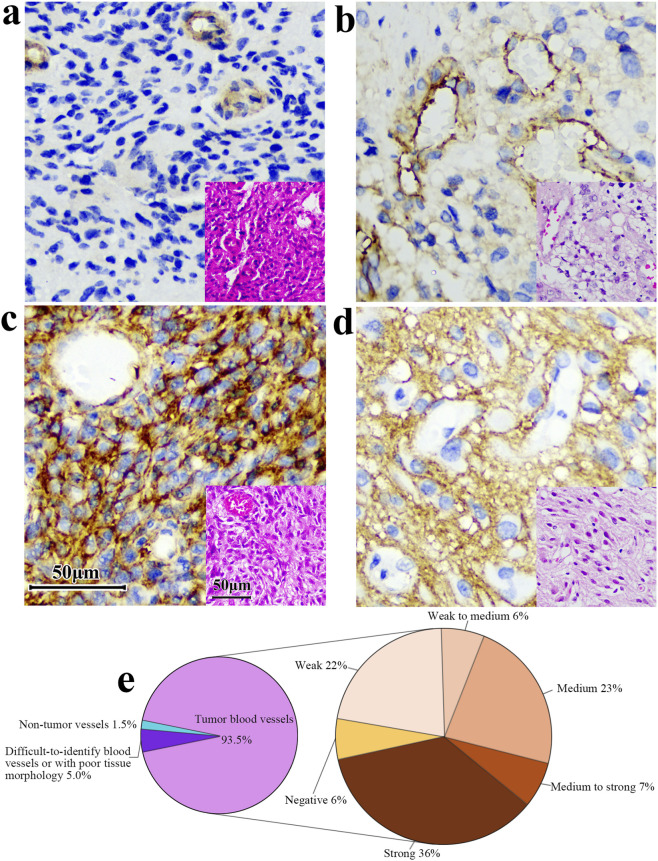
The B7-H3 protein exhibits differential expression levels in the vasculature of gliomas. **(a)** Vessel positive, surrounding tissue negative. **(b,c)** Both vessels and surrounding tissue are positive. **(d)** Vessels are negative while surrounding tissues are positive. Corresponding HE at right corner of each image. Scale bar 50 μm. Original magnification, ×400. **(e)** Among the 200 samples with positive B7-H3 protein detection, 93.5% of the tumor blood vessels and 1.5% of the non-tumor blood vessels were observed. Additionally, the blood vessels in 5% of the samples were difficult to identify or had poor tissue morphology. Among the tumor blood vessels, the proportions of different intensities of B7-H3 protein expression were as follows: 6% were negative, 22% were weakly positive, 6% were from weakly positive to moderately positive, 23% were moderately positive, 7% were from moderately positive to strongly positive, and 36% were strongly positive.

Intriguingly, upon microscopic examination of IHC slides, we found that 85.4% of the blood vessels in gliomas were positive for B7-H3 expression (176/206, Figure 9). In a cohort of 200 samples with positive B7-H3 protein expression, 5% (10 cases) of the samples’ blood vessels were either difficult to identify or exhibited poor tissue morphology and were consequently excluded from the interpretation scope. 93.5% (187 cases) were identified as tumor blood vessels. Among these, 94% (176 cases) of the blood vessels expressed B7-H3 protein. We stratified the positive degree into five gradations, namely, weakly positive (Pale yellow), weakly positive to moderately positive (Between pale yellow and dark yellow), moderately positive (Dark yellow), moderately positive to strongly positive (Between dark yellow and dark brown), and strongly positive (Dark brown). The specific proportions are illustrated in Figure 9. The staining intensity of blood vessels did not display a perfectly congruent correlative pattern with the overall slide interpretation results. The microscopic examination images are presented in Figure 9. We used Anti - VEGF Receptor 2 antibody to label blood vessels in glioma tissues and 36H7 to identify B7-H3 protein (Supplementary Figure S7). The coincidence of the two fluorescences indicated that in the blood vessels, the B7-H3-positive cells are vascular endothelial cells.

## 4 Discussion

Immunohistochemistry (IHC) is a widely used detection method in pathology and serves as an important tool for clinical diagnosis. Physicians typically evaluate a patient’s status based on the staining patterns observed in tissue slides to guide therapeutic decisions. However, scoring criteria are non-standardized, and and the absence of harmonized evaluation systems presents challenges for consistent interpretation.

Among the three scoring approaches evaluated in this study, each offers distinct advantages and limitations. The Tumor Proportion Score (TPS) assesses only the percentage of positive tumor cells and does not incorporate staining intensity. As such, it emphasizes the presence of the target antigen while potentially underestimating the biological significance of expression strength. The H-score integrates staining intensity into a semi-quantitative framework; however, it may amplify subjective variation among pathologists and obscure spatial or qualitative differences. For example, two slides could yield an identical H-score of 30, one derived from 30% weak (1^+^) staining and the other from 10% strong (3^+^) staining, masking the difference between low- and high-intensity staining patterns.

Conversely, classification based solely on the percentage of 2^+^ and 3^+^ positive cells excludes tumors with 1^+^ staining and should be applied only when evidence indicates that such cases derive no therapeutic benefit. When appropriate, however, this approach enables more refined stratification, particularly for strongly stained tumors (e.g., 50%–100% positivity), which may be less clearly differentiated using an H-score.

The B7-H3 (36H7) antibody was developed using a single B cell antibody screening approach, which significantly shortened the production timeline while yielding high-affinity candidates. The identified heavy and light-chain sequences were subsequently cloned into expression vectors and transiently transfected into HEK293 cells for recombinant production, resulting in enhanced antibody stability.

Notably, antibodies targeting B7-H3 (CD276) display distinct epitope specificities. The 36H7 clone recognizes an extracellular domain, where intercellular interactions typically occur via cell surface molecules for signal transduction. In contrast, clones SP265 and SP206 bind to an intracellular epitope (amino acids 488–534), rendering them undetectable by flow cytometry without membrane permeabilization (Supplementary Figure S8). The clone D9M2L recognizes a region near amino acid Ala94 of the B7-H3 protein. Similar to the B7-H3(36H7) antibody, it targets the extracellular domain.

As a diagnostic-class antibody, B7-H3 (36H7) was designed to exhibit not only high specificity but also performance characteristics suitable for clinical use, including thermal stability, stability during transport, and a prolonged shelf life. When used as a companion diagnostic to guide therapy-particularly CAR-T cell treatment-its immunohistochemical results must be correlated with both treatment safety and efficacy. Beyond IHC, the antibody also holds potential for applications in flow cytometry and ELISA-based diagnostic kits.

In addition to its general staining, we observed that a significant proportion of glioma samples exhibit B7-H3 expression in blood vessels, a finding that was corroborated using the D9M2L clone (data not shown). Previous studies have reported that B7-H3 is expressed at higher levels in the blood vessels of various tumor tissues compared with normal vessels (Mesri et al., 2013), highlighting its active role in angiogenesis. Additionally, B7-H3 protein promotes the proliferation of human endothelial cells and the secretion of vascular endothelial growth factor (VEGF), thereby regulating angiogenesis and migration (Lai et al., 2019). Consistent with this, our simultaneous detection of B7-H3 and VEGF fluorescence in blood vessels (Supplementary Figure S7) supports the association of B7-H3 expression with endothelial cell proliferation and vascular formation. Therapeutically, blocking the interaction between B7-H3 and its receptor may represent a strategy to inhibit tumor neovascularization and, consequently, tumor growth.

As previously described, B7-H3 (36H7) could be employed as a companion diagnostic reagent, guiding treatment decisions for patients who meet the predefined biomarker threshold. However, the clinical utility of B7-H3 as a biomarker extends beyond this application. A retrospective analysis of 86 isocitrate dehydrogenase wild-type (IDH wt) glioblastoma patients treated with surgery, radiotherapy, and temozolomide revealed that high B7-H3 expression was significantly associated with poorer overall survival (OS) and progression-free survival (PFS) (Yüceer et al., 2025). These findings suggest that clinical detection of B7-H3 protein expression may assist physicians in comprehensive patient management throughout the treatment course. Similarly, in papillary thyroid carcinoma (PTC), Bohui Zhao et al. examined 343 postoperative whole-tumor slides and detected B7-H3 positivity in 84.8% of PTC cases (291/343). Their observations indicate that B7-H3 serves as a novel independent biomarker for predicting lymph node metastasis (LNM) and disease recurrence in PTC patients (Zhao et al., 2022). These findings suggest that clinical detection of B7-H3 can support comprehensive patient management and risk-adapted treatment strategies.

While next-generation sequencing (NGS) offers high sensitivity and throughput, its implementation imposes substantial cost burdens for both healthcare institutions and patients. Furthermore, NGS data analysis requires 3–7 days, whereas IHC detection can typically be completed within 24 h, offering a faster, simpler, and more cost-effective method for detecting protein-level gene expression. Importantly, IHC with the 36H7 antibody allows direct visualization of spatial protein distribution within tissues, facilitating assessment of the tumor microenvironment. Unlike NGS, which is dependent on nucleic acid quality and susceptible to mRNA degradation during long-term storage, paraffin-embedded tissues stored at −20 °C show minimal antigen loss within 3 months.

With the continuous progress of biomarker and targeted therapy research, antibodies such as 36H7 will play an increasingly important role in personalized medicine, guiding treatment decisions and improving patient prognosis.

## 5 Conclusion

We have developed a new B7-H3 antibody clone with high affinity for its target epitope. Using this antibody, we established a quality control program with tonsil tissue as the reference. The excellent specificity, stability of this antibody facilitate glioma diagnosis and provide guidance for treatment.

## Data Availability

The original contributions presented in the study are included in the article/Supplementary Material, further inquiries can be directed to the corresponding author.

## Supplementary material

SUPPLEMENTARY FIGURE S1 B7-H3 staining on lung adenocarcinoma samples of **(a)** D9M2L(concentration: 0.2 μg/mL), **(b)** 36H7(concentration: 1.0 μg/mL) and **(c)** SP206(concentration: 0.5 μg/mL), **(e)** 36H7(concentration: 0.2 μg/mL),**(f)** SP206(concentration: 0.2 μg/mL). **(d)** is corresponding HE. When the concentrations of the three antibodies were set to 0.2 μg/mL, the 36H7 **(e)** and SP206 **(f)** clones unable to effectively recognize adenocarcinoma cells(shows a trabecular arrangement in a ring-like pattern). Each antibody clone requires its own optimal staining conditions. Original magnification, ×400.

SUPPLEMENTARY FIGURE S2 B7-H3 staining on tonsil tissues of **(a)** D9M2L, **(b)** 36H7 and **(c)** SP206, **(d)** Isotype rabbit IgG. Immunohistochemical analysis of tonsil tissue revealed differential staining intensities among the three antibody clones evaluated. scale bar 50 μm. Original magnification, ×400.

SUPPLEMENTARY FIGURE S3 This antibody is stable and can be stored in a wide range of conditions. The concentration of the B7-H3(36H7) antibody is 1.0 μg/mL. All of the slides Staining by BOND III. **(a)** Staining of normal brains before testing was negative. **(b)** The result remains negative after five freeze-thaw cycles of the antibody. **(c)** No non-specific adsorption after 7 days of heating the antibody. **(d)** Staining of glioma before testing was positive. The antibody can still recognize the target after freeze-thaw **(e)** and heating **(f)**. scale bar 50 μm. Original magnification, ×400.

SUPPLEMENTARY FIGURE S4 The fluorescent signals produced by the two antibodies overlap, all CD21-positive FDCs express B7-H3 protein in germinal center(arrow). **(a)** CD21-mIF (Coral Pink), **(b)** CD21-IHC, **(c)** Corresponding HE, **(d)** Merge, **(e)** B7-H3-mIF (green), **(f)** nuclei. scale bar 100 μm.

SUPPLEMENTARY FIGURE S5 B7-H3 protein was detected in all eight tumor samples. The B7-H3 protein is expressed in a variety of tumor tissues, and the B7-H3 (36H7) antibody can be effectively recognized on slides of other tumor types. **(a)** gastric adenocarcinoma. **(b)** lung adenocarcinoma. **(c)** hepatocellular carcinoma. **(d)** breast - non-special invasive breast cancer. **(e)** cervix - squamous cell carcinoma. **(f)** prostate – adenocarcinoma. **(g)** kidney - clear cell carcinoma. **(h)** glioma. scale bar 50 μm. Original magnification, ×400.

SUPPLEMENTARY FIGURE S6 B7-H3 staining of the tissue microarray (TMA). Results of IHC experiments with B7-H3 (36H7) antibody on 37 healthy human organs dots. Strong staining: Endometrium (A1), Liver (A2), Pancreas (A3). Partial weak staining: Placenta (A4), Prostate (A5), Bladder (A6), Eye (A7), Parathyroid (B1), Testis (B2), Adrenal gland (B3), Esophagus (B4), salivary gland (B5), Pituitary (B6), Nerve peripheral (C1), Fallopian tube (C2), Lung (C3), Intestine colon (C4), Skin (C5), Uterus cervix (C6), Tonsil (D1, Black arrow), Spleen (D2), Small intestine (D3), rectum (D4), Thymus (D5). Negative: Brain (D6), Bone marrow (E1), Straited muscle (E2), Spinal cord (E3), Thyroid (E4), Ovary (E5), Breast (E6), Kidney cortex (F1), Kidney medulla (F2), Ureter (F3), Stomach (F4), Heart (F5), cerebellum (F6) scale bar 1 mm.

SUPPLEMENTARY FIGURE S7 The fluorescence signal of B7-H3 protein overlapped with the fluorescence signal of the endothelial cell marker VEGF, indicating that the expression of B7H3 protein in endothelial cells was positive **(a)** merge, **(b)** VEGF, **(c)** cell nucleus, **(d)** B7-H3.

SUPPLEMENTARY FIGURE S8 Flow cytometry was performed on B7-H3-expressing U251 cells using three monoclonal antibodies: 36H7 (orange), SP265 (blue), and SP206 (purple), with PBS as a negative control (cyan). Only 36H7, which targets the extracellular domain, showed positive staining. SP265 and SP206, recognizing intracellular epitopes, failed to bind without membrane permeabilization.
