# Supplementary figures and images for: Development and characterization of a novel B7-H3 rabbit monoclonal antibody for glioma diagnosis
Source: Front Pharmacol. 2026 Jan 23;17:1736583. doi: 10.3389/fphar.2026.1736583 (PMC12876247; doi:10.3389/fphar.2026.1736583)

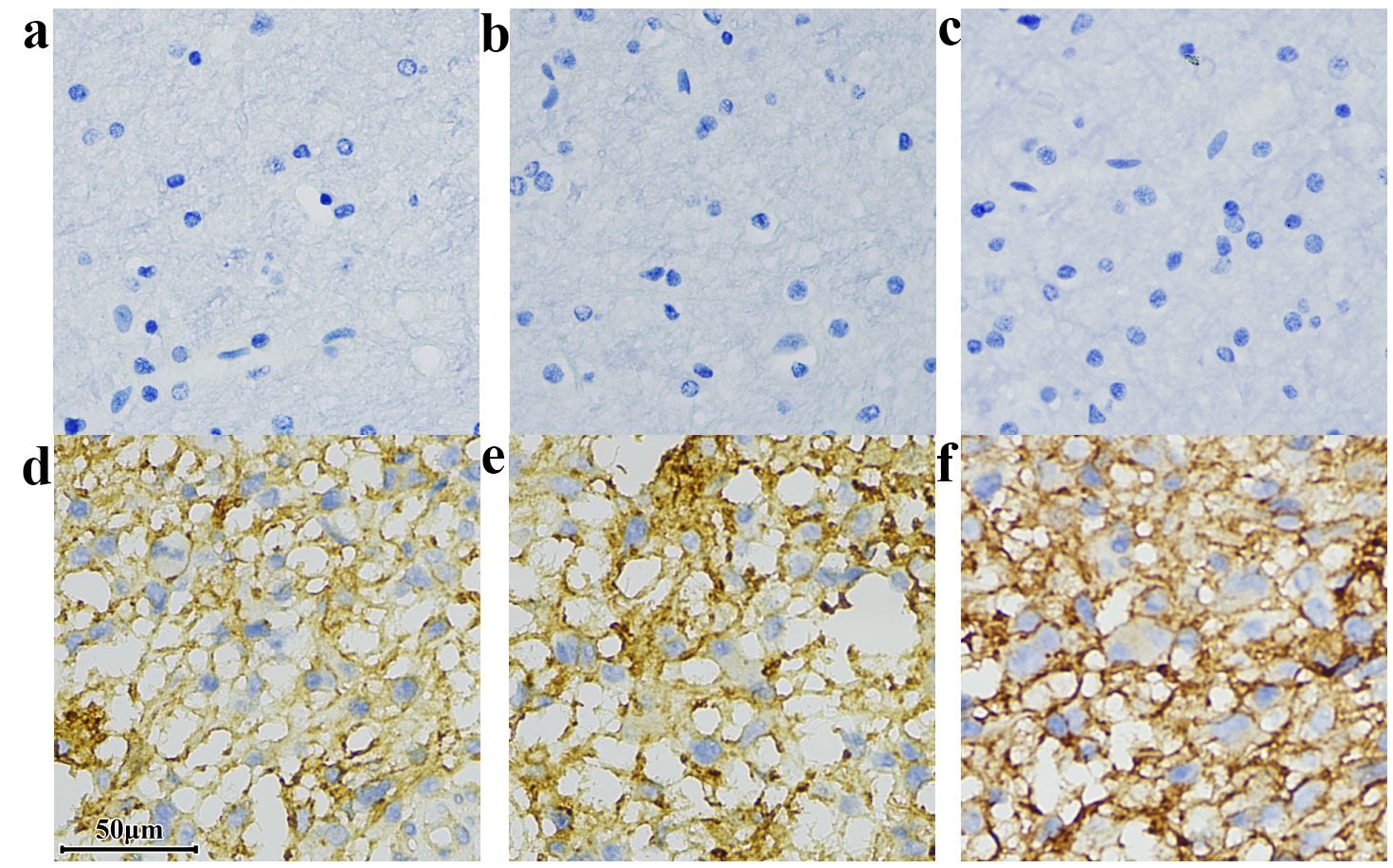

Supplement: Supplementary file 1 [file Image3.jpeg]

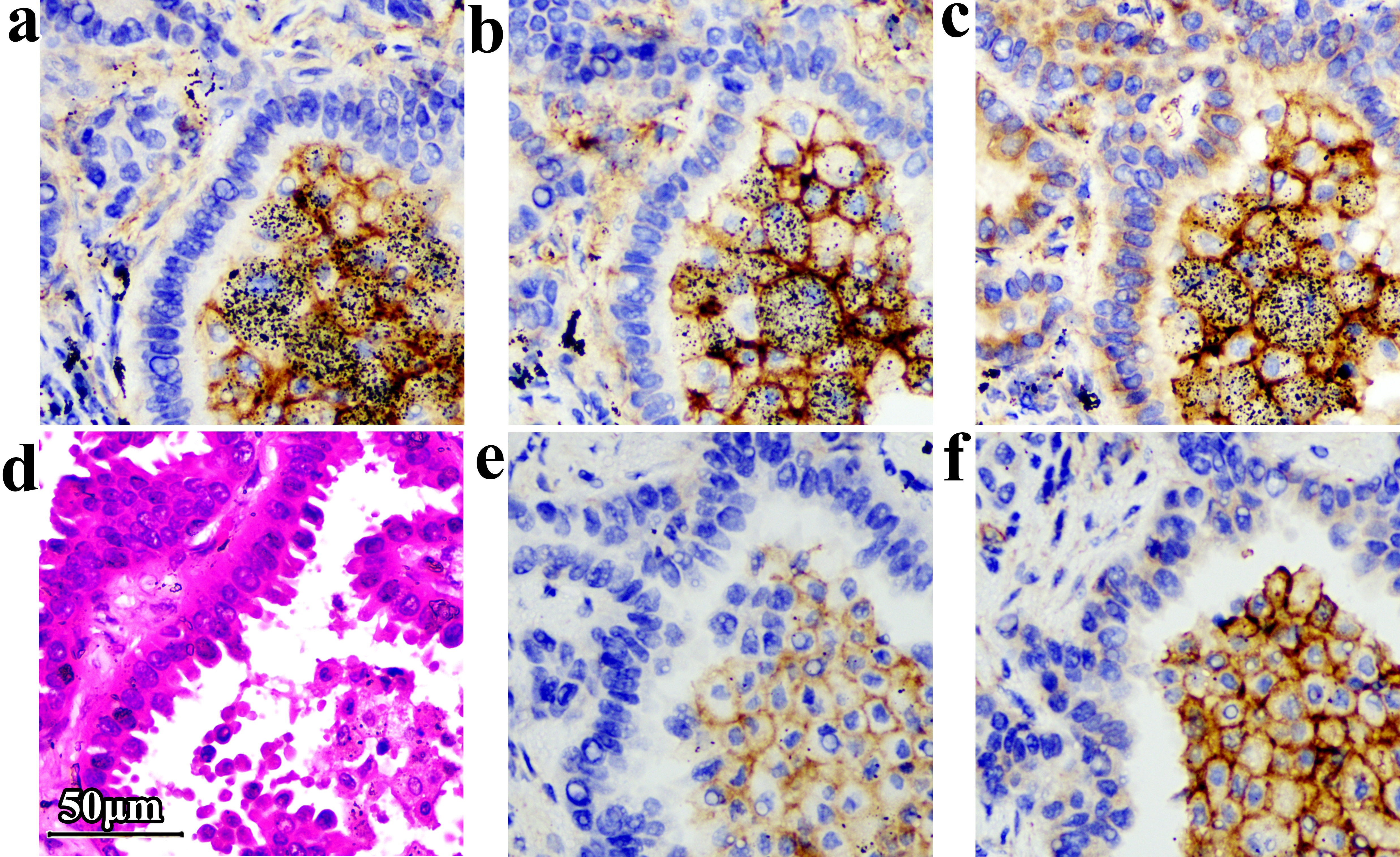

Supplement: Supplementary file 3 [file Image1.jpeg]

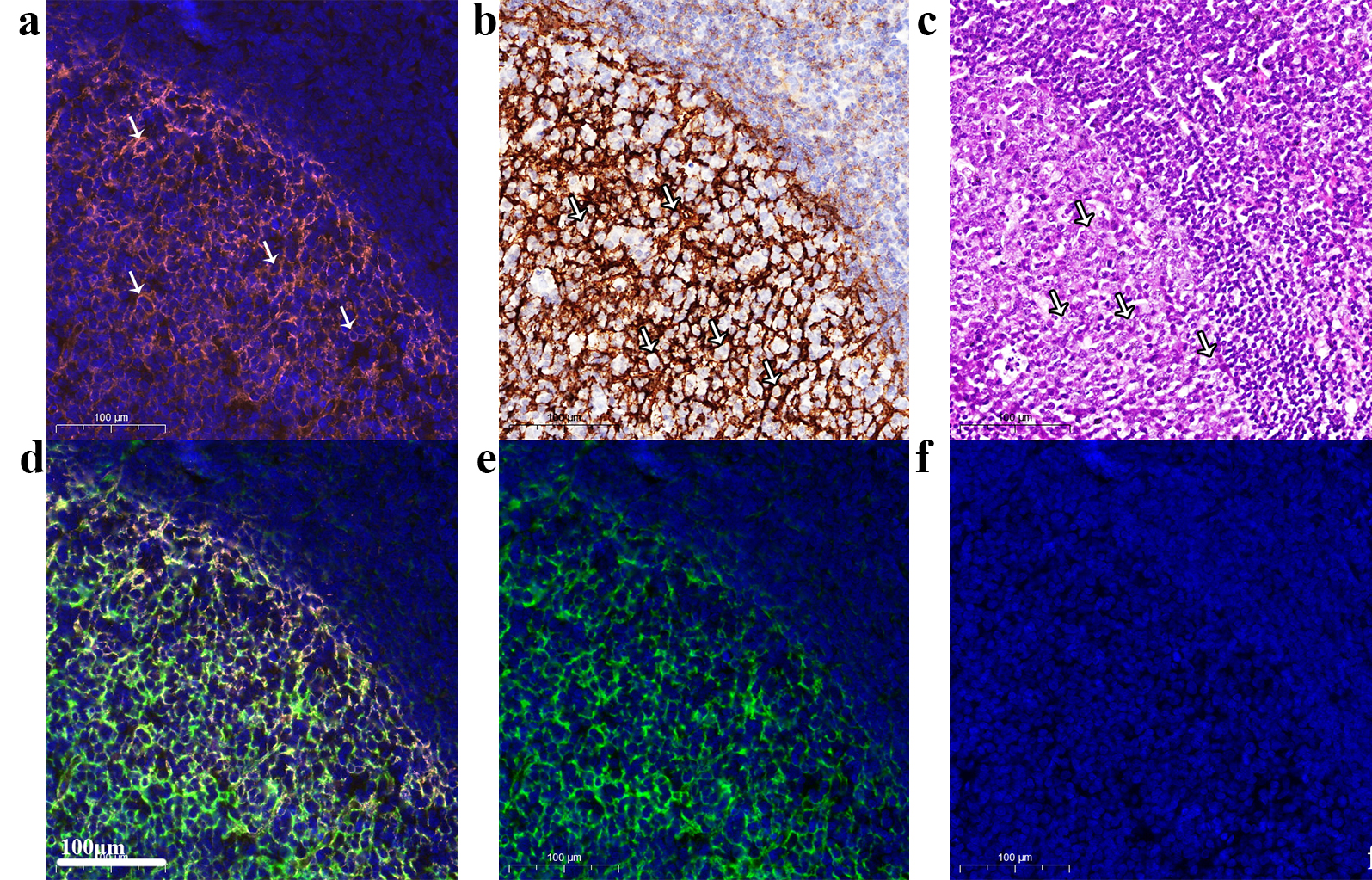

Supplement: Supplementary file 4 [file Image4.jpeg]

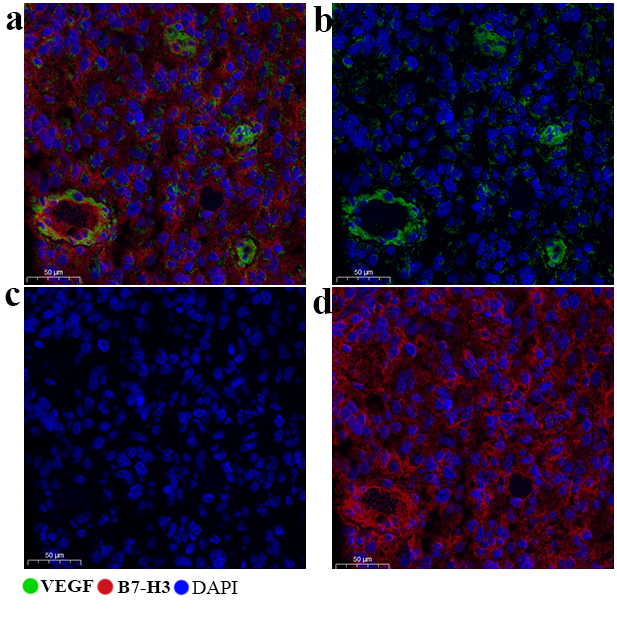

Supplement: Supplementary file 5 [file Image7.jpeg]

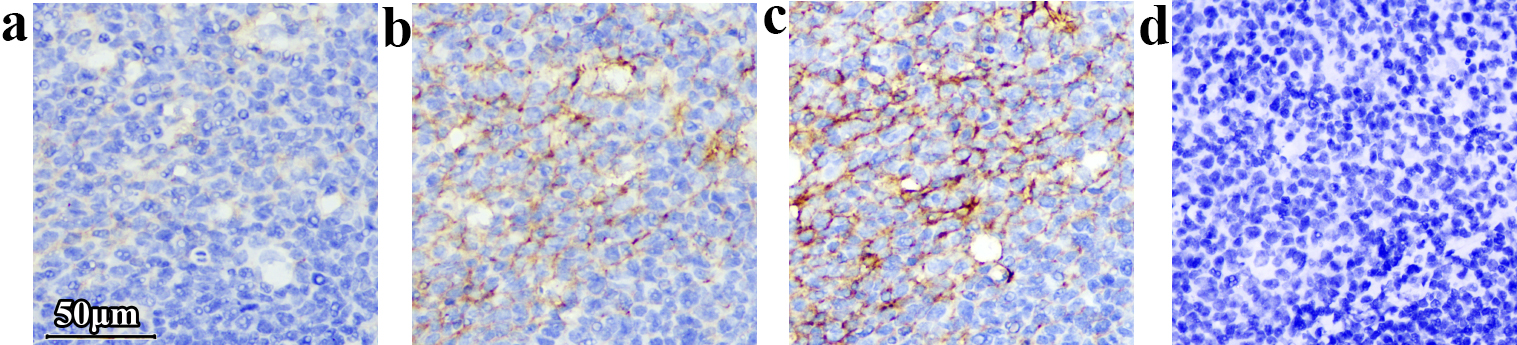

Supplement: Supplementary file 6 [file Image2.jpeg]

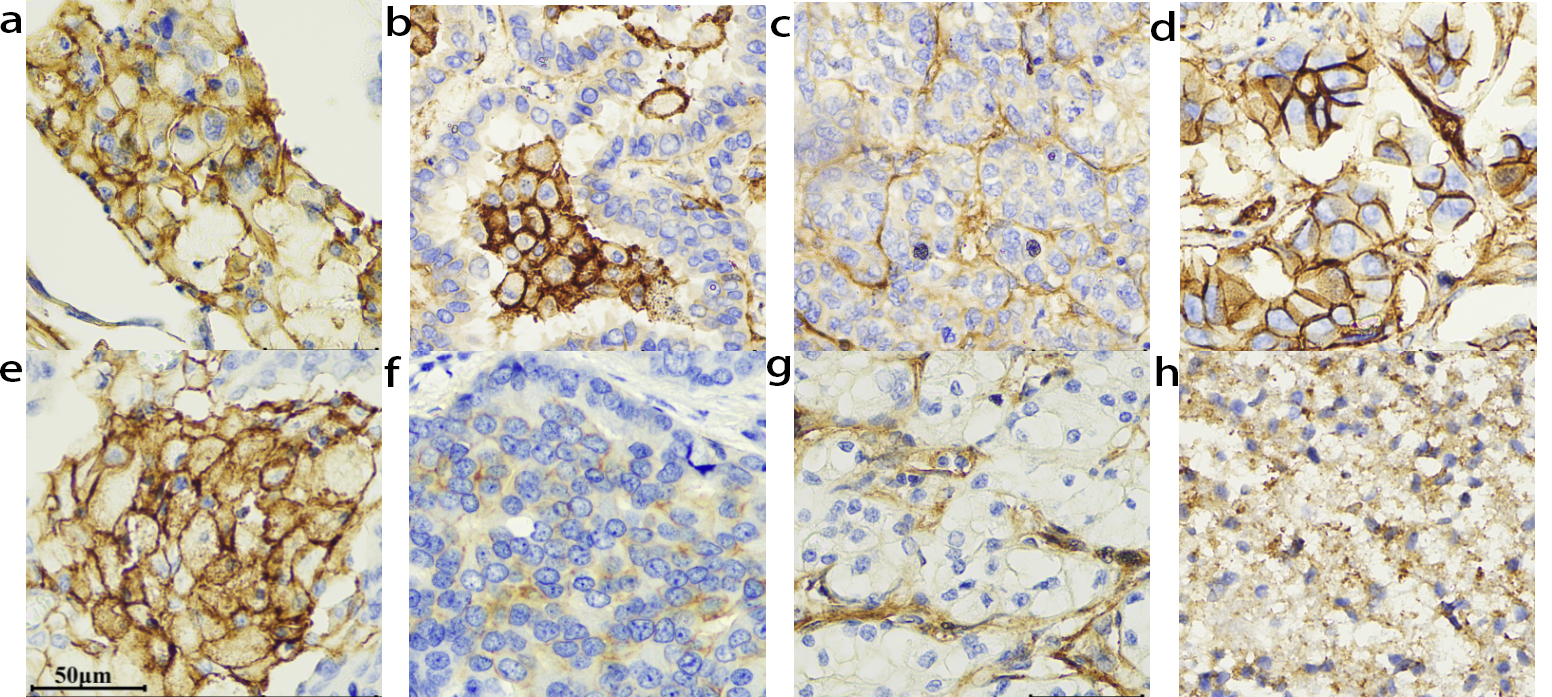

Supplement: Supplementary file 7 [file Image5.jpeg]

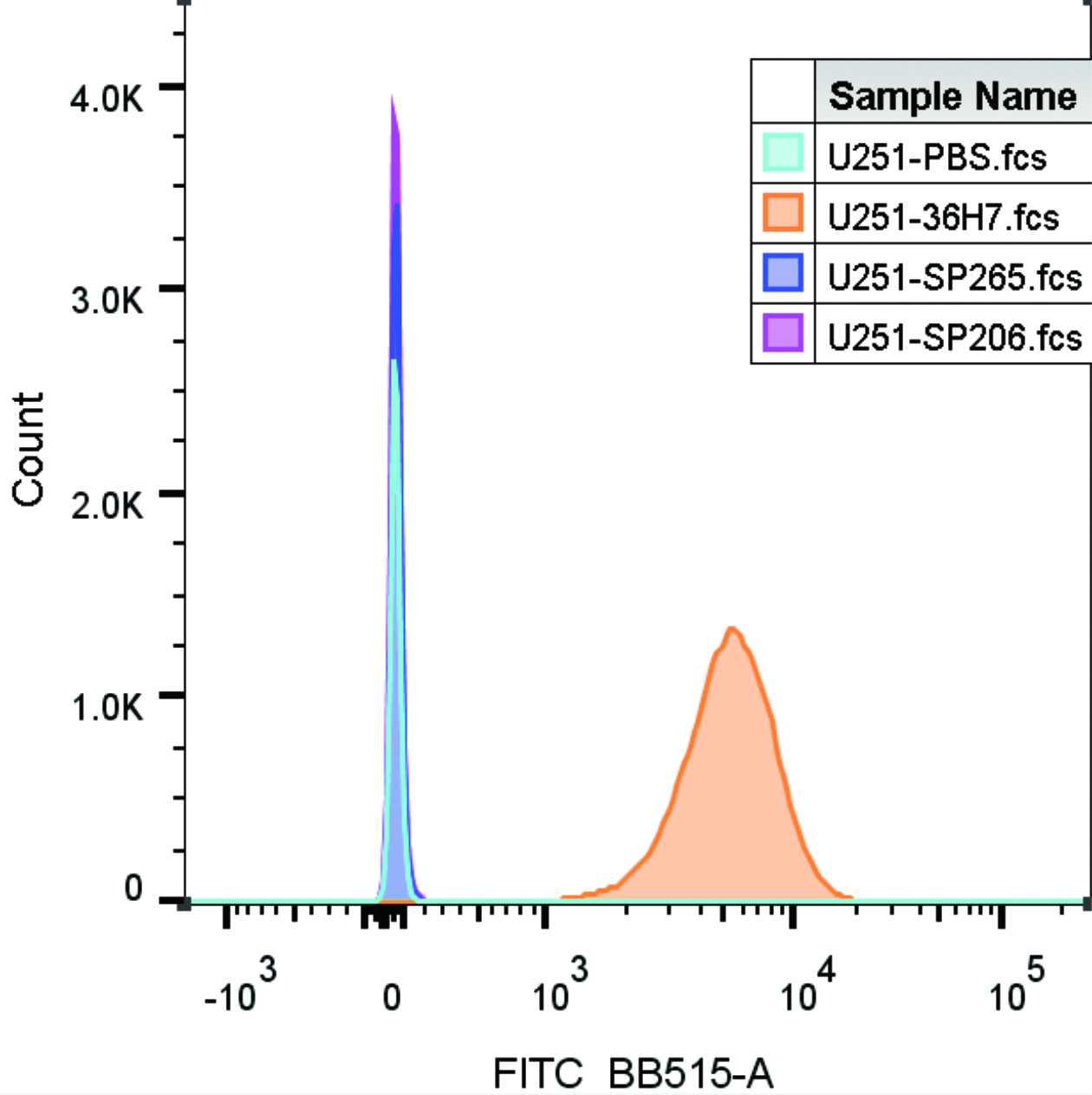

Supplement: Supplementary file 8 [file Image8.jpeg]

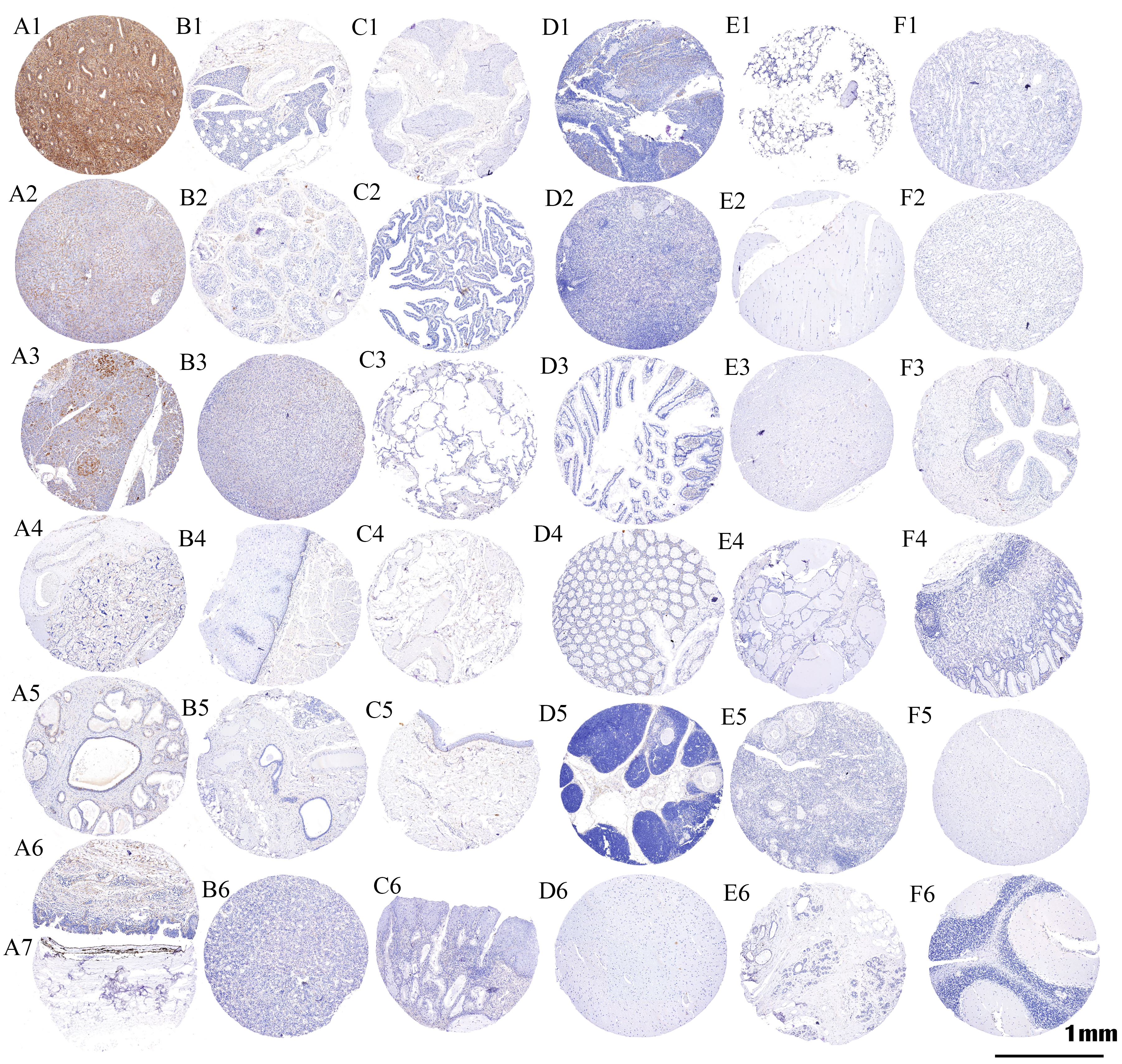

Supplement: Supplementary file 9 [file Image6.jpeg]
