# Supplementary material for: Development and characterization of a novel B7-H3 rabbit monoclonal antibody for glioma diagnosis
Source: Front Pharmacol. 2026 Jan 23;17:1736583. doi: 10.3389/fphar.2026.1736583 (PMC12876247; doi:10.3389/fphar.2026.1736583)
Supplement: Supplementary file 2 [file Table1.docx]

| **Supplement table 1.**  The percentile of B7-H3-positive cells (slides=50) | | | |
| --- | --- | --- | --- |
| Areas | The percentile of B7-H3-positive cells | Variable coefficient | P value |
| Germinal center | 61.5±8.9%* | 0.144 | a<0.001 |
| Crypt epithelium | 47.2±12.7% | 0.270 | b<0.001 |
| Interfollicular | 1.8±2.3% | 1.281 |  |

1. Germinal center contrasts interfollicular, b. Crypt epithelial contrasts interfollicular

* The average positivity rate of 50 slides ± SD
